# Detection and Quantification of Viable Mycobacterium tuberculosis Bacilli in Saline-Processed Stool Samples by Tuberculosis Molecular Bacterial Load Assay: a Potential Alternative for Processing Stool

**DOI:** 10.1128/spectrum.00274-22

**Published:** 2022-05-12

**Authors:** Emmanuel Musisi, Abdul Ssesolo, Derek J. Sloan, Stephen H. Gillespie, Wilber Sabiiti

**Affiliations:** a Division of Infection and Global Health, School of Medicine, University of St. Andrews, St. Andrews, United Kingdom; b Infectious Diseases Research Collaboration, Kampala, Uganda; c Department of Biochemistry and Sports Sciences, Makerere University, Kampala, Uganda; Texas A&M University

**Keywords:** diagnosis, *Mycobacterium tuberculosis*, phosphate-buffered saline, RNA, stool, tuberculosis, tuberculosis molecular bacterial load assay, viable bacillary load

## LETTER

Pulmonary tuberculosis (PTB) is the major form of active tuberculosis (TB) disease, and diagnosis mainly depends on detection of Mycobacterium tuberculosis in sputum (1, 2). However, pediatric patients and patients with advanced HIV struggle to produce sputum (3). Studies have also shown that sputum productivity decreases as patients progress past 2 months of treatment. The need for an alternative sample type to diagnose TB cannot be more strongly emphasized. We have shown that viable M. tuberculosis bacilli are quantifiable by the TB molecular bacterial load assay (TB-MBLA) in stool samples processed using OMNIgene-Sputum (OM-S) medium (4).

OM-S, which is manufactured by DNA Genotek (Canada), preserves the viability of M. tuberculosis and suppresses contaminants in sputum, enabling samples to be transported farther without requiring cold chain conditions (5, 6). In this letter, we provide data to demonstrate that phosphate-buffered saline (PBS), a widely used laboratory reagent, is a potential alternative sample-processing medium for stool-based diagnosis of TB.

A set of stool samples corresponding to those processed in the OM-S study were processed using PBS and stored at −20°C until RNA extraction was performed (4). Six grams of stool per patient was processed within 20 min after collection, prior to storage. Bacillary loads were measured by TB-MBLA and compared to those of OM-S-processed stool samples. Prior to freezing, mycobacterial growth indicator tube (MGIT) culture was performed, and contamination rates for the two stool-processing methods were determined. Stool TB-MBLA sensitivity and specificity were calculated using sputum MGIT culture as a reference test.

Stool samples from 100 presumptive cases were analyzed, of which 61 (61%) were confirmed to be PTB positive by sputum MGIT culture (Table 1). TB-MBLA positivity for PBS-processed stool samples was 53% (53/100 samples), 4% less than the value for OM-S-processed stool samples from presumptive cases. Positivity was 77% (47/61 samples) for cases confirmed for TB by MGIT culture. The average bacillary load was 4.28 ± 0.95 log_10_ estimated CFU/mL in PBS-processed stool samples, on average 0.8 log_10_ eCFU/mL less than the load detected in OM-S-processed stool samples (Mann-Whitney test, *P* = 0.003). TB-MBLA sensitivity and specificity were 77% (95% confidence interval [CI], 65 to 87%) and 87% (95% CI, 73 to 96%), respectively, and were consistent with those for OM-S-processed stool samples. The TB-MBLA positive predictive value for PBS-processed stool samples was 92%, 6% higher than that for OM-S-processed stool samples. The MGIT culture contamination rate of 35% for PBS-processed stool samples was 23% higher than that for OM-S-processed stool samples (Table 2).

**TABLE 1:** Demographic and clinical characteristics of study participants

Characteristic^*a*^	Data for participants with PTB status of^*b*^:			*P* ^*c*^
	Overall (*n* = 100)	Positive (*n* = 61)	Negative (*n* = 39)	
Age (median [IQR]) (yr)	34 (25–42)	33 (25–41)	36 (26–45)	0.72
Female (no. [%])	53 (53)	32 (52.5)^*d*^	21 (53.9)^*e*^	0.8
HIV-positive (no. [%])	36 (35)	20 (33)^*d*^	16 (41)^*e*^	0.27
ART use (no. [%])	20 (38)	10 (16.4)^*d*^	10 (26)^*e*^	0.31
CD4^+^ cell count (median [IQR]) (cells/mm^3^)^*f*^	110 (44–228)	71 (26–171)	170 (66–254)	0.03

a IQR, interquartile range; ART, antiretroviral therapy.

b Bacteriologically confirmed positive or negative cases.

c Comparison between PTB-positive and PTB-negative participants.

d Percentage of bacteriologically confirmed TB cases.

e Percentage of bacteriologically confirmed TB-negative cases.

f Measured for HIV-infected participants only (*n *= 36).

**TABLE 2:** Comparative performance of TB-MBLA and MGIT culture with PBS-processed versus OM-S-processed stool samples

Parameter	Data for:		*P*
	OM-S-processed stool samples (*n* = 100)	PBS-processed stool samples (*n* = 100)	
Confirmed PTB by MGIT sputum culture (no. [%])	61 (61)	61 (61)	
Positive by stool TB-MBLA only (no. [%])	8 (8)	4 (4)^*a*^	
Positive by both MGIT sputum culture and stool TB-MBLA (no. [%])^*b*^	49 (49)	47 (47)^*a*^	
Bacterial load (mean ± SD) (log_10_ estimated CFU/mL)^*c*^	5.1 ± 1.59	4.28 ± 0.95	0.003
Threshold cycle (median [IQR])	20 (15–25)	22 (21–25)	0.002
Stool contamination by MGIT culture (no. [%])	26 (26)	69 (69)	
Stool contamination by MGIT culture but TB-MBLA positive (no. [%])	12 (46)	35 (51)	
Sensitivity (% [95% CI])	80 (68–89)	77 (65–87)	
Specificity (% [95% CI])	79 (63–90)	90 (76–97)	
Positive predictive value (% [95% CI])	86 (74–93)	92 (81–98)	
Negative predictive value (% [95% CI])	72 (56–85)	71 (57–83)	

a Forty-seven samples were sputum MGIT culture-stool TB-MBLA positive, while 4 samples were stool TB-MBLA positive only. Overall stool TB-MBLA positivity was 51% (51/100 samples) or 77% (47/61 samples) considering sputum MGIT culture as the gold standard.

b Sputum MGIT was used as the gold standard and reference test for TB-MBLA.

c Bacterial load values were log transformed before the mean was calculated.

The findings show an ~1-log-unit decrease in quantifiable bacterial load in PBS-processed stool samples, compared to OM-S-processed stool samples. This could be explained by the fact that the TB-MBLA was performed on stool samples that had been stored at −20°C for more than 1 year, conditions under which the M. tuberculosis RNA-preserving ability might have been lower than that of OM-S. This means that PBS-processed stool samples might achieve similar sensitivity, compared to OM-S-processed samples, if TB-MBLA is performed with freshly prepared stool samples.

OM-S was previously shown to be a strong preservative of M. tuberculosis, as well as suppressing non-M. tuberculosis contaminants (5). However, TB-MBLA uses primers and probes specific to M. tuberculosis and is not affected by non-M. tuberculosis contaminants found in patient sputum (7). This eliminates the need to use decontaminating reagents to process stool samples or other samples for TB diagnosis using molecular tests such as TB-MBLA; we previously showed that such processes reduce the viable count by 0.6 log_10_ CFU/mL on average (8). Based on these findings, we think that PBS may be an effective and inexpensive alternative for the preparation of stool samples for TB-MBLA and other molecular applications in both resource-rich and resource-limited settings. Larger studies are needed to verify the performance of PBS in recovering viable M. tuberculosis bacilli from both fresh and frozen stool samples, compared to the established RNA-preserving reagents.

### Data availability.

Raw data will be available at the University of St Andrews upon request and meeting of the ethical requirements according to which the samples were collected.
